# Precision therapeutics for early onset epilepsy: Allele-selective antisense oligonucleotides for SCN2A-related epilepsy

**DOI:** 10.1016/j.omtn.2026.103068

**Published:** 2026-09-07

**Authors:** Olivia Kim-McManus

**Affiliations:** 1 Department of Neurosciences, University of California, San Diego, La Jolla, CA, USA; 2 Rady Children’s Health, San Diego, CA, USA

## Main text

Developmental and epileptic encephalopathies (DEE) frequently present with uncontrolled seizures from birth, and SCN2A is among the most prevalent monogenic epilepsies, with pathogenic variants accounting for 1%–2% of all epileptic encephalopathies.^1^ Many causal variants confer gain-of-function or mixed-function effects—increased channel open probability or elevated persistent sodium current—and genotype-phenotype relationships within the gene are correspondingly heterogeneous.^2^^,^^3^ Conventional anti-seizure medications and surgical neuromodulatory approaches can confer meaningful therapeutic benefit in patients with DEE^4^: nonetheless seizure control remains challenging in many patients, and developmental trajectories are typically static or slowly progressive despite optimized pharmacotherapy.^2^ Targeted genetic therapy directed at the underlying molecular etiology, if identified, may therefore provide additional benefit.

Individualized antisense oligonucleotide (ASO) therapy has established precedent in nano-rare disease, beginning with milasen in CLN7 neuronal ceroid lipofuscinosis^5^ and extending to SCN2A-DEE through elsunersen, a non-allele-selective ASO administered to a preterm infant under expanded access.^6^

Per recent publication in *Nature Medicine* which was featured in *Nature Research Highlight*, we conducted two parallel open-label, single-center, single-patient (n-of-1) trials in two male patients, aged 9 and 14 years, each harboring a distinct heterozygous pathogenic SCN2A variant: c.5645G>A (p.Arg1882GLn), a gain-of-function variant, and c.2558G>A (p.Arg853GLn), a mixed gain- and loss-of-function variant.^1^^,^^7^ Rather than targeting the causal mutations, allele-selective ASOs were designed against patient-specific heterozygous intronic single-nucleotide polymorphisms (SNPs) lying in *cis* with each proband’s pathogenic variant, enabling discrimination between mutant and wild-type transcripts independent of the mutation itself.^1^ Each ASO was manufactured as a single-patient investigational agent under the nano-rare regulatory framework established for such programs,^8^^,^^9^ with protocol and dose-escalation schedule individualized to each participant (NCT06314490).^10^

ASO was administered by intrathecal lumbar injection at approximately 2–3 months intervals over 16–24 months. Safety and tolerability were assessed longitudinally alongside seizure frequency, standardized developmental and functional measures, including the Bayley Scales of Infant and Toddler Development and phenotype-specific instruments.^1^

Both participants tolerated investigational therapeutic intervention with no ASO-related adverse events or serious adverse events. There were no clinically significant changes in hematological or biochemical parameters and electrocardiography relative to baseline.^1^ Both demonstrated reductions in seizure burden, although the magnitude and statistical robustness of the response differed. In one study participant, the reduction in seizure frequency did not reach statistical significance, but the accompanying clinical improvement was sufficient to permit discontinuation of phenytoin—a sodium channel blocker on which he had been dependent since birth—indicating benefit layered on top of an already partially effective regimen. The other study participant showed a marked reduction, with sustained seizure-free intervals of weeks to months and acquisition of independent ambulation at age 15 years, a milestone not achieved before intervention. Meaningful gains in quality of life, communication, motor skills, and maladaptive behavior were seen.^1^

Several conclusions follow. First, allele-selective design using variant-linked SNPs rather than the pathogenic sequence is a generalizable strategy: it worked across two unrelated genotypes and extends in principle to any gene with private, heterozygous, gain-of-function variants. Notably, the two probands’ tag SNPs had population allele frequencies of 0.27 and 0.18, implying that a single haplospecific ASO may serve patients well beyond its index case—the premise of a companion analysis proposing diplotype-based matching of pre-qualified ASOs to newly diagnosed patients.^11^ Second, the safety profile observed here, consistent with prior single-patient SCN2A experience,^6^ supports repeated intrathecal ASO administration as a durable modality in this population. Third, the developmental gains—de novo independent ambulation in a 15-year old—are of a magnitude unlikely to reflect natural history in a population characterized by static or worsening trajectories. Fourth, participants were treated after years of established disease and nonetheless acquired therapeutic benefit. Because the genetic cause is identifiable by neonatal genome sequencing, and intrathecal ASO delivery is tolerable in early infancy,^6^ substantially earlier intervention is achievable; these responses in heavily affected, long-symptomatic patients may be a conservative estimate of what earlier treatment might yield.

The therapeutic logic—allele-selective suppression of a transcript with a net excitatory consequence—was applied to one gain-of-function and one mixed variant; predominantly loss-of-function SCN2A variants are mechanistically distinct and require an orthogonal, augmentation-based approach. Individualized manufacturing and regulatory pathways, academic infrastructure and expertise for delivery remain resource-intensive and require funding support although haplospecific approach theoretically enables scalability.^11^

Mechanistically informed, allele-selective ASO therapy can be safely designed, manufactured, and administered to patients with rare SCN2A variants, yielding clinically meaningful seizure and developmental outcomes in addition to those provided by conventional anti-seizure medications and surgical neuromodulatory approaches—an early but structurally important step toward ASO therapeutics as a treatment paradigm for rare neurogenetic disease, including developmental and epileptic encephalopathies.

## Acknowledgments

Funding: O.K.-M. discloses related research grant support from 10.13039/100000900California Institute for Regenerative Medicine (CIRM CLIN2-15085).

## Declaration of interests

The author(s) and immediate family members have no competing interests to declare.
